# Combined multidirectional plyometric and balance training enhances neuromuscular, postural and sport-specific performance in soccer players: a randomized controlled trial

**DOI:** 10.3389/fphys.2026.1848877

**Published:** 2026-07-01

**Authors:** Yazeed Nawahda, Thierry Paillard, Jamal Abubshara, Roland van den Tillaar, Mohamed Chedly Jlid

**Affiliations:** 1 Research Laboratory (LR23JS01) Sport Performance, Health and Society, Higher Institute of Sport and Physical Education of Ksar Said, University of Manouba, Manouba, Tunisia; 2 Université de Pau et des Pays de l’Adour, UPPA, MEPS, Tarbes, France; 3 Palestine Technical University – Kadoorie, Tubas and Northen Valleys, Palestine; 4 Department of Sport Sciences and Physical Education, Nord University, Levanger, Norway

**Keywords:** agility, balance training, neuromuscular performance, plyometric training, soccer, unstable surface

## Abstract

This study examined the effects of combining multidirectional plyometric training with balance exercises performed on an unstable surface on neuromuscular performance, dynamic postural control, inter-limb balance asymmetry, and sport-specific motor skills in U17 soccer players. Forty-five male youth soccer players (16–17 years) were randomly assigned to a combined plyometric and balance training group (n = 15), a plyometric training group (n = 15), or a control group (n = 15). The intervention lasted 8 weeks, with three sessions per week. Assessments included squat jump, countermovement jump, drop jump, 10-, 20-, and 30-m sprint, reactive agility, Y-Balance Test performance, inter-limb asymmetry, slalom dribble, and wall-pass tests. Both experimental groups improved significantly in sprint, jump, dynamic balance, and sport-specific motor skill performance compared with the control group (p < 0.05). No significant between-group differences were found for jump performance. Reactive agility improved significantly more in the combined group than in the plyometric group. Dynamic postural control improved in both training groups, with greater gains in the combined group in the anterior and posteromedial directions for both dominant and non-dominant legs. Inter-limb asymmetry decreased in all three directions in the combined group, whereas the plyometric group improved only in the anterior and posterolateral directions. In sport-specific motor skills, both training groups improved, although no significant difference was found between the plyometric and control groups in the wall-pass test. Combined training appears to provide additional benefits for reactive agility, selected balance measures, inter-limb asymmetry and sport-specific skills in youth soccer players.

## 1 Introduction

Soccer is a high-intensity intermittent sport characterized by repeated bouts of sprinting, high-speed running, jumping, accelerations, decelerations, and rapid changes of direction combined with technical actions such as dribbling, passing, and shooting (Curtis et al., 2018; Gualtieri et al., 2023). These movements place substantial neuromuscular and physiological demands on players and require well-developed strength, power, agility, and postural control to optimize performance (Curtis et al., 2018; Gualtieri et al., 2023). Consequently, enhancing these physical capacities has become a key objective of conditioning programs in youth soccer players (Slimani et al., 2016; Sarmento et al., 2018).

Among the various training strategies used to improve athletic performance in soccer, plyometric training has been widely recognized as an effective method for enhancing explosive strength and neuromuscular performance (Jlid et al., 2019; Jlid et al., 2020). Plyometric exercises improve the efficiency of the stretch–shortening cycle, allowing athletes to produce greater force in a shorter period of time during explosive actions such as sprinting and jumping (Markovic and Mikulic, 2010). Previous studies have consistently demonstrated that plyometric training can significantly enhance vertical jump performance, sprint ability, and change-of-direction performance in soccer players (Chelly et al., 2010; Ramírez-Campillo et al., 2014). Recent systematic reviews have reported that plyometric training is an effective strategy for enhancing explosive performance and sprint ability in youth and team-sport athletes (Slimani et al., 2016; Kons et al., 2023).

Because soccer performance involves movements performed in multiple planes, training programs incorporating multidirectional exercises may better replicate the biomechanical and neuromuscular demands of the sport (Jlid et al., 2019; Jlid et al., 2020). Multidirectional plyometric training integrates vertical, horizontal, and lateral movements, enabling athletes to develop explosive strength and neuromuscular control in several directions while reproducing key soccer actions such as cutting, accelerating, and landing. Previous research has shown that multidirectional plyometric training can improve vertical jump performance, change-of-direction ability, and dynamic postural control in soccer players (Jlid et al., 2019; Jlid et al., 2020).

Dynamic postural control represents another crucial component of soccer performance (Zech et al., 2010; Paillard, 2023; Giustino et al., 2024). During match play, players frequently perform complex movements while maintaining stability on a single limb during actions such as kicking, landing, or cutting. Therefore, improvements in dynamic balance and neuromuscular control may not only enhance athletic performance but also contribute to reducing the risk of lower-limb injuries (Zech et al., 2010; Paillard, 2023).

Despite the growing body of literature examining the effects of plyometric training in soccer players, most previous studies have primarily focused on traditional or unidirectional plyometric exercises (Markovic and Mikulic, 2010; Ramírez-Campillo et al., 2014). In contrast, the potential benefits of combining multidirectional plyometric training with balance exercises performed on unstable surfaces remain insufficiently explored, particularly in youth soccer players (Behm and Colado, 2012; Gonzalo-Skok et al., 2017). This approach may be novel and practically relevant because unstable-surface balance training increases proprioceptive and sensorimotor demands, while multidirectional plyometric training targets explosive force production and the stretch-shortening cycle. Combining these stimuli may therefore better reflect sport-specific actions that require players to produce force while maintaining postural stability during unilateral landings, cutting maneuvers, accelerations, decelerations, and ball-related actions. Furthermore, limited research has investigated whether such combined training strategies may influence soccer-specific motor skills and inter-limb balance asymmetry (Bishop et al., 2018b; Dos’ Santos et al., 2018). Evidence suggests that sport-specific motor skills are important determinants of performance in soccer players, whereas inter-limb balance asymmetry may negatively affect motor performance, including power and agility, and may increase the risk of lower-extremity injury (Bishop et al., 2018b; Brown et al., 2018).

Thus, the aim of this study was to examine the effects of combining multidirectional plyometric training with balance exercises performed on an unstable cushion on the overall performance of young soccer players, including neuromuscular performance, dynamic postural control, inter-limb balance asymmetry, and sport-specific motor skills. We hypothesized that the combined intervention would induce greater improvements than plyometric training alone because it simultaneously challenges explosive force production, proprioceptive regulation, postural stability, and unilateral movement control, all of which are required during multidirectional soccer actions.

## 2 Materials and methods

### 2.1 Participants

Forty-five male youth soccer players from clubs competing in the Palestinian national league volunteered to participate in this study. Participants were recruited through their clubs after coaches and club staff had been informed about the aims and procedures of the study. Eligible players were then invited to participate and received verbal and written information about the study procedures, potential risks, and benefits before providing written informed consent. Participants were aged between 16 and 17 years and were regular members of their U17 teams. Inclusion criteria were male youth soccer player aged 16–17 years, regular participation in club training and competition, and availability to complete the full 8-week intervention and testing schedule. Exclusion criteria were any hip, knee, ankle, lower-limb, or lower-back injury requiring medical treatment or causing interruption of training or match participation during the previous three months; any current musculoskeletal disorder affecting the lower limbs or lower back; or any medical condition contraindicating maximal exercise testing or plyometric training. An *a priori* sample size calculation was performed using G*Power software (version 3.1). The analysis assumed an alpha level of 0.05, a statistical power of 0.95, and an effect size of 0.25, indicating that a minimum sample of 36 participants was required. To account for potential dropouts during the intervention period, 45 players were recruited. Participants were randomly assigned, using a computer-generated randomization sequence with a 1:1:1 allocation ratio, to one of three groups: a combined plyometric and balance training group (n = 15), a plyometric training group (n = 15), and a control group (n = 15).

### 2.2 Experimental design

This study was designed as a three-arm, parallel-group randomized controlled trial with a 1:1:1 allocation ratio and was reported in accordance with the CONSORT 2010 statement (Schulz et al., 2010). The study protocol was approved by the Ethics Committee of the Deanship of Scientific Research at Palestine Technical University-Kadoorie. All participants received both verbal and written information regarding the study procedures, potential risks, and benefits before providing written informed consent in accordance with the Declaration of Helsinki. Participants were also informed that they were free to withdraw from the study at any time without any consequences.

The intervention lasted eight weeks and was conducted during August and September. During this period, the combined group performed a combined training program including multidirectional plyometric exercises and balance exercises on unstable surfaces. The plyometric group completed the same multidirectional plyometric training program without balance exercises, while the control group continued their regular soccer training routine without any additional training intervention (**Tables 1**, **2**).

**Table 1:** Plyometric training program (Group 2) per training session over the 8-week intervention.

Week	Training 1 (vertical)	Training 2 (lateral)	Training 3 (anterior-posterior)	Total ground contacts
Content	Sprinter power step-up & vertical box jumps;	Lateral barrier jumps & skater hops	Forward-backward hurdle jumps & standing broad jumps	
1	3 Sets × 6 Reps	3 Sets × 6 Reps	3 Sets × 6 Reps)	108
2	3 Sets × 6 Reps	3 Sets × 6 Reps	3 Sets × 6 Reps	108
3	4 Sets × 6 Reps	4 Sets × 6 Reps	4 Sets × 6 Reps	144
4	4 Sets × 6 Reps	4 Sets × 6 Reps	4 Sets × 6 Reps	144
5	5 Sets × 6 Reps	5 Sets × 6 Reps	5 Sets × 6 Reps	180
6	5 Sets × 6 Reps	5 Sets × 6 Reps	5 Sets × 6 Reps	180
7	6 Sets × 6 Reps	6 Sets × 6 Reps	6 Sets × 6 Reps	216
8	6 Sets × 6 Reps	6 Sets × 6 Reps	6 Sets × 6 Reps	216

15s rest between reps, 60s rest between sets.

**Table 2:** Plyometric and balance training program over the 8-week intervention.

Week	Training 1 (vertical)	Training 2 (lateral)	Training 3 (anterior-posterior)	Total volume (plyo contacts / total balance)
Content	Vertical box jumps + sprinter step-ip & single-leg balance on air cushion	Lateral barrier jumps + skater hops & proprioceptive reach on air pad.	Forward–backward hurdle jumps + standing broad jumps & forward lunge on air cushion.	
1	2 Sets × 6 Reps + Balance 3×30s	2 Sets × 6 Reps + Balance 3×30s	2 Sets × 6 Reps + Balance 3×30s	24 Contacts / 9 Min
2	2 Sets × 6 Reps + Balance 3×30s	2 Sets × 6 Reps + Balance 3×30s	2 Sets × 6 Reps + Balance 3×30s	24 Contacts / 9 Min
3	3 Sets × 6 Reps + Balance 3×30s	3 Sets × 6 Reps + Balance 3×30s	3 Sets × 6 Reps + Balance 3×30s	36 Contacts / 12 Min
4	3 Sets × 6 Reps + Balance 3×30s	3 Sets × 6 Reps + Balance 3×30s	3 Sets × 6 Reps + Balance 3×30s	36 Contacts / 12 Min
5	4 Sets × 6 Reps + Balance 3×30s	4 Sets × 6 Reps + Balance 3×30s	4 Sets × 6 Reps + Balance 3×30s	48 Contacts / 15 Min
6	4 Sets × 6 Reps + Balance 3×30s	4 Sets × 6 Reps + Balance 3×30s	4 Sets × 6 Reps + Balance 3×30s	48 Contacts / 15 Min
7	5 Sets × 6 Reps + Balance 3×30s	5 Sets × 6 Reps + Balance 3×30s	5 Sets × 6 Reps + Balance 3×30s	60Contacts / 18 Min
8	5 Sets × 6 Reps + Balance 3×30s	5 Sets × 6 Reps + Balance 3×30s	5 Sets × 6 Reps + Balance 3×30s	60 Contacts / 18 Min

15s rest between jumps, 15s rest between balance reps, and 60s rest between sets.

All measurements were conducted in the same indoor testing area at the participating club’s sports facility. The evaluations were carried out over four consecutive days under similar experimental conditions. Initial and final measurements were performed at the same time of day, between 4:00 and 6:00 p.m., in order to minimize the potential influence of diurnal variation on physical performance (Chtourou et al., 2012). Players were instructed to avoid strenuous physical activity 24 h before each testing session. On Day 1, anthropometric measurements (body height, body mass, and leg length) were obtained, followed by vertical jump assessments including the squat jump, countermovement jump, and drop jump to evaluate lower-limb explosive power. On Day 2, participants performed sprint tests over distances of 10 m, 20 m, and 30 m to assess acceleration and sprint performance, in addition to a reactive agility test. On Day 3, dynamic postural control was assessed using the Y-Balance Test, with reach distances in the anterior, posteromedial, and posterolateral directions normalized to leg length and expressed as a percentage. On Day 4, sport-specific motor skills were evaluated using two field-based tests: the slalom dribble test and the wall-pass test, which were designed to assess technical performance.

### 2.3 Testing procedures

#### 2.3.1 Anthropometric measurements

Body height was measured using a wall-mounted stadiometer (Seca 216, Hamburg, Germany). Body mass was assessed with participants barefoot and wearing light clothing using a calibrated digital scale (EKS Focus 9800, Gislaved, Sweden). Leg length was measured by the same experienced investigator as the distance from the anterior superior iliac spine to the distal tip of the lateral malleolus (Filipa et al., 2010) using a standard stainless-steel tape measure, with participants lying in a supine position on a plinth. Leg dominance was determined according to van Melick, Meddeler, Hoogeboom, Nijhuis-van der Sanden and van Cingel (van Melick et al., 2017) Body mass index (BMI) was calculated as body mass (kg) divided by height squared (m^2^).

#### 2.3.2 Vertical jump performance

Vertical jump performance was assessed using three tests: the squat jump, countermovement jump, and drop jump. All jumps were performed using an infrared photocell system (Optojump, Microgate^®^, Bolzano, Italy), which measures flight time to estimate jump height. This system has previously demonstrated acceptable validity and reliability for assessing vertical jump performance (Lehance et al., 2009).

For the squat jump, participants started from a static semi-squat position with approximately 90° knee flexion while keeping their hands on their hips to eliminate arm swing. After briefly holding this position, participants performed a maximal vertical jump without any preparatory countermovement, following the procedure described by Ghoul, Tabben, Miarka, Tourny, Chamari and Coquart (Ghoul et al., 2019). For the countermovement jump, participants began from an upright standing position with their hands placed on their hips. They then performed a rapid downward movement by flexing the hips and knees to approximately 90° knee flexion, followed immediately by a maximal vertical jump. For the drop jump, participants stepped off a 30-cm box and landed on the contact platform before immediately performing a maximal vertical jump. Participants were instructed to minimize ground contact time and jump as high as possible, as commonly described in plyometric assessment protocols (Markovic and Mikulic, 2010). Three trials were performed for each jump condition, and the best performance was retained for statistical analysis. Test–retest reliability demonstrated high consistency, with intraclass correlation coefficients (ICC) of ≥0.93 for all jumps, with 95% confidence intervals (CI) ranging from 0.88 to 0.97.

#### 2.3.3 Sprint performance (10 m, 20 m and 30 m)

Before the sprint assessments, participants completed a standardized 20-minute warm-up. Sprint performance was then evaluated through maximal sprints over distances of 10 m, 20 m, and 30 m from a stationary starting position. Sprint times were recorded using paired photoelectric timing gates (Microgate, Bolzano, Italy) positioned at 10 m, 20 m, and 30 m. Each participant performed three sprint trials with a recovery period of 5–6 minutes between attempts to minimize fatigue. The fastest time recorded for each distance was retained for further analysis. Test–retest reliability demonstrated excellent consistency, with intraclass correlation coefficients (ICC) of 0.81 for 10-m, 0.84 for 20-m and 0.92 for 30-m split times, and 95% confidence intervals ranging from 0.83 to 0.95.

#### 2.3.4 Reactive agility test

**Figure 1:**
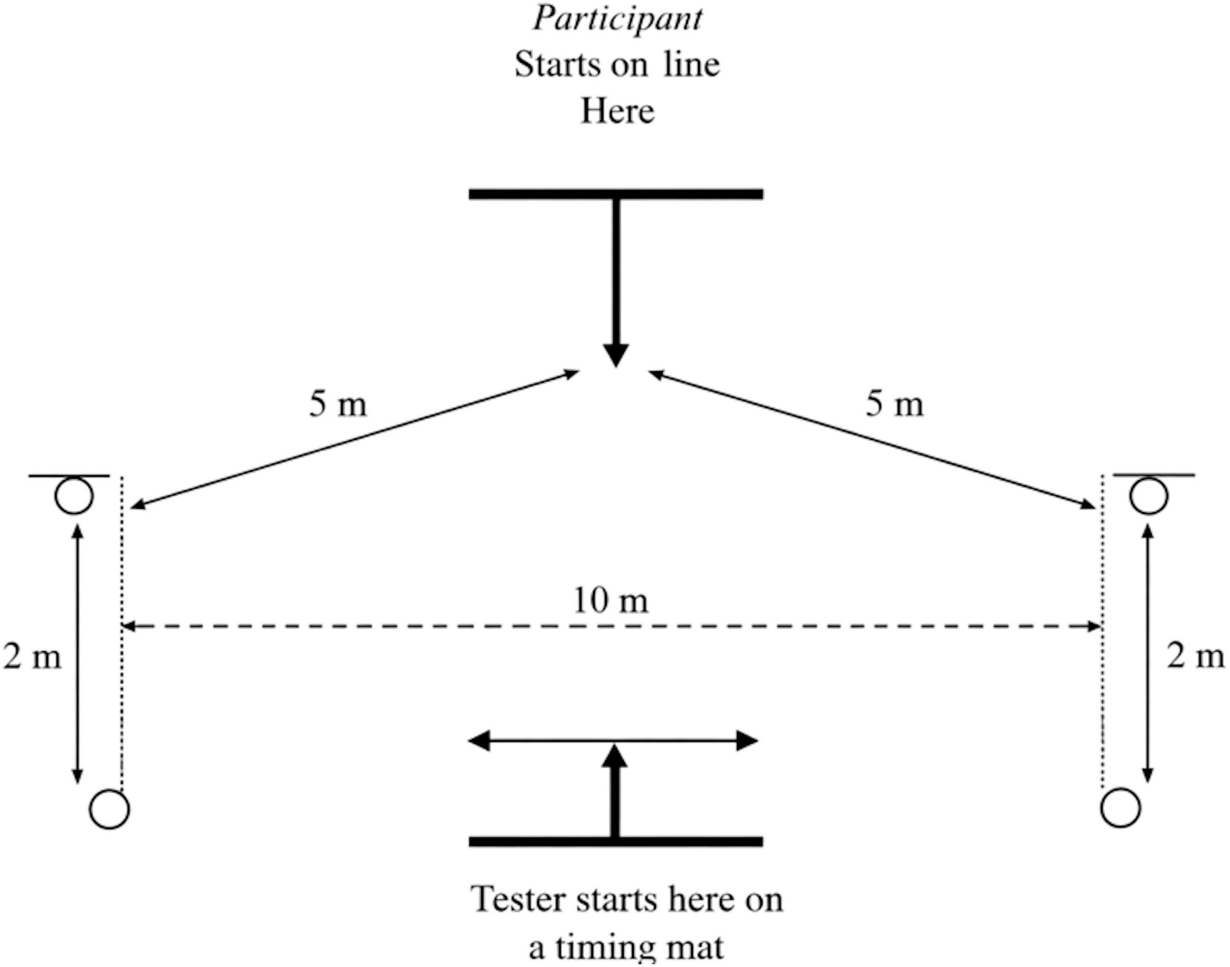
Reactive agility test.

Reactive agility was assessed using the Reactive Agility Test described by Sheppard and Young (2006) (**Figure 1**). Participants started on a marked line. Timing gates were positioned 5 m to the left and right and 2 m in front of the start line, resulting in an inter-gate distance of 10 m. The tester stood opposite the participant on a timing mat connected to the timing-gate software. When the tester stepped off the timing mat, an audible signal was emitted and the timing commenced. Each participant performed three trials, with adequate recovery between attempts to minimize fatigue, and the fastest time was retained for statistical analysis. Test–retest reliability demonstrated excellent consistency, with intraclass correlation coefficients (ICC) of 0.91 and 95% confidence intervals ranging from 0.85 to 0.94.

#### 2.3.5 Dynamic postural control (Y-Balance Test)

Dynamic postural control was assessed using the Y-Balance Test, which is a reliable and valid tool for evaluating dynamic balance and lower-limb stability in athletes (Plisky et al., 2009; Shaffer et al., 2013). The Y-Balance Test is a simplified version of the Star Excursion Balance Test and measures reach distance in three directions: anterior, posteromedial, and posterolateral. Participants stood barefoot on one leg at the center of the Y-shaped testing grid while reaching with the contralateral leg in each direction. They were instructed to maintain balance on the stance leg while reaching as far as possible with the free limb without losing balance or lifting the stance foot. Each participant performed three trials in each direction for both lower limbs. Reach distances were recorded and normalized to leg length to account for anthropometric differences using the following formula:

*Normalized reach distance (%) = (reach distance/leg length) × 100*

The best score obtained in each direction was retained for statistical analysis. Test–retest reliabilities (ICC) for the three reach directions ranged from 0.92 to 0.97 for the dominant leg (95% CI: 0.95–0.98, 0.94–0.98, 0.88–0.95) and from 0.95 to 0.97 for the non-dominant leg (95% CI: 0.95–0.98, 0.92–0.97, 0.92–0.97).

#### 2.3.6 Dynamic balance asymmetry

Dynamic balance asymmetry was calculated using the normalized reach distances obtained from the Y-Balance Test. Inter-limb asymmetry was determined by comparing the performance of the dominant and non-dominant legs for each reach direction (anterior, posteromedial, and posterolateral). Asymmetry values were calculated using the following formula (Bishop et al., 2018a):

*Asymmetry (%) = |dominant − non-dominant|/dominant × 100*

This method allows the identification of potential imbalances between limbs, which may be associated with decreased performance and increased injury risk in athletes.

#### 2.3.7 Sport-specific motor skills

**Figure 2:**
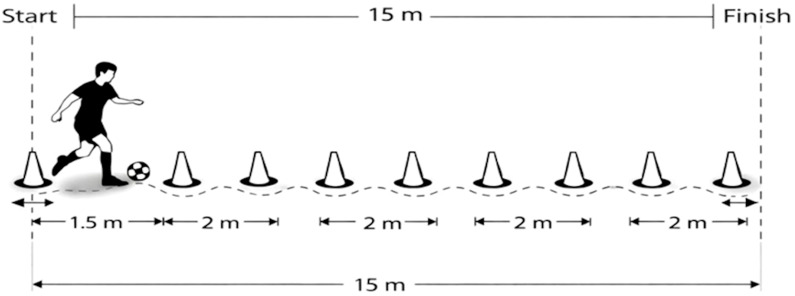
Slalom dribble test.

**Figure 3:**
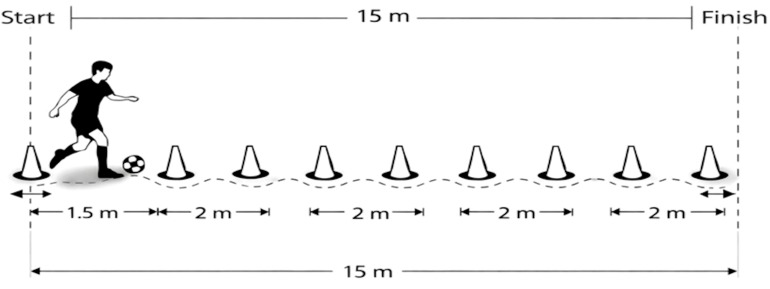
McDonald Soccer Skill Test.

Sport-specific motor skills were assessed using two field-based soccer tests: the slalom dribble test and the wall-pass test. The slalom dribble test was performed according to Padrón-Cabo, Rey, Kalén and Costa (Padrón-Cabo et al., 2020) (**Figure 2**). Participants completed a maximal dribble through seven cones arranged over 15 m, including a 1.5-m sprint to the first cone, a slalom through six cones spaced 2 m apart, and a final 1.5-m sprint to the finish line. A 3-min recovery interval was provided between trials, and the test was performed with the dominant foot. The wall-pass test was based on the McDonald Soccer Skill Test described by Kapre, Alexander and Palekar (Kapre et al., 2025) (**Figure 3**). A 30-ft-wide and 11-ft-high kickboard was placed in front of the participant. The starting line was set 9 ft from the kickboard, and a restraining line was positioned 18 ft away, with two additional footballs placed behind it. Participants were instructed to strike the kickboard as many times as possible within 30 s while maintaining ball control. If the rebound was insufficient, they could either retrieve the same ball or use one of the additional balls. Three trials were completed for each test, and the best performance was used for statistical analysis. Test-retest reliability was high, with ICC values of 0.90 for the zigzag dribbling test and 0.91 for the wall-pass test (95% CI: 0.84-0.94).

### 2.4 Training program

The training intervention lasted eight weeks and was implemented three times per week (Sunday, Tuesday, and Thursday) as part of the participants’ regular training schedule. Participants in the plyometric group performed a plyometric training program, whereas those in the combined group performed plyometric training combined with balance exercises on an unstable cushion. All training sessions were supervised by members of the research team, with each researcher responsible for one group. Before the intervention, all researchers involved in supervision were familiarized with the training protocols to ensure standardized exercise instruction, correct execution, adherence to the training program, and participant safety. Each training session included multidirectional plyometric exercises organized into three movement categories: vertical, lateral, and anterior-posterior power exercises. The same research team supervised both the plyometric and balance components. Each training session included multidirectional plyometric exercises organized into three movement categories: vertical, lateral, and anterior-posterior power exercises. In the combined training group, additional balance exercises were performed on an unstable cushion to further challenge balance control and proprioception. The recovery period between repetitions within each set was approximately 15 s (Read and Cisar, 2001), while a rest interval of about 60 s was allowed between sets. The duration of the training sessions increased progressively over the intervention period, ranging from 30 to 75 minutes. Detailed information regarding the weekly training volume and the total number of ground contacts for the plyometric training group is presented in **Table 1**, while the combined training program, including both plyometric and balance exercises, is described in **Table 2**. It should be noted that although the plyometric component was similar across the two experimental groups, the combined group also performed balance exercises; therefore, the total training stimulus differed between experimental groups.

### 2.5 Statistical analysis

All data are presented as mean ± 95% confidence intervals (CI). The normality of data distribution was verified using the Shapiro-Wilk test. Test-retest reliability was assessed using intraclass correlation coefficients (ICCs). A two-way repeated-measures analysis of variance (ANOVA) was performed to examine the effects of group (combined training group, plyometric training group, and control group) and time (pre-intervention vs. post-intervention) on the measured variables. When significant interactions or main effects were detected, Holm-Bonferroni *post hoc* tests were applied to identify pairwise differences. Effect sizes (ES) were calculated using Cohen’s d. According to Cohen (1988), effect sizes were interpreted as small (0.00 ≤ *d* ≤ 0.49), medium (0.50 ≤ *d* ≤ 0.79), or large (*d* ≥ 0.80). Statistical significance was set at p < 0.05. All statistical analyses were conducted using version 0.95.4. of JASP (University of Amsterdam, Amsterdam, Netherlands).

## 3 Results

All data followed a normal distribution. Baseline anthropometric and descriptive characteristics were comparable across the three groups, with no significant between-group differences observed at baseline (p > 0.05; **Table 3**).

**Table 3:** mean ± SD age and anthropometric characteristics of participants.

Variable	Combined group(n = 15)	Plyometric group (n = 15)	Control group (n = 15)	ANOVA p
Age (years)	16.13 ± 0.35	16.33 ± 0.48	16.20 ± 0.41	0.424
Body height (cm)	173.60 ± 3.99	173.73 ± 6.81	172.27 ± 4.80	0.709
Leg length (cm)	91.93 ± 3.15	90.73 ± 4.78	90.27 ± 4.35	0.531
Body mass (kg)	64.73 ± 4.83	63.80 ± 4.16	61.60 ± 4.27	0.151
Body mass index (kg·m^-^²)	21.50 ± 1.80	21.18 ± 1.58	20.81 ± 1.98	0.575

**Figure 4:**
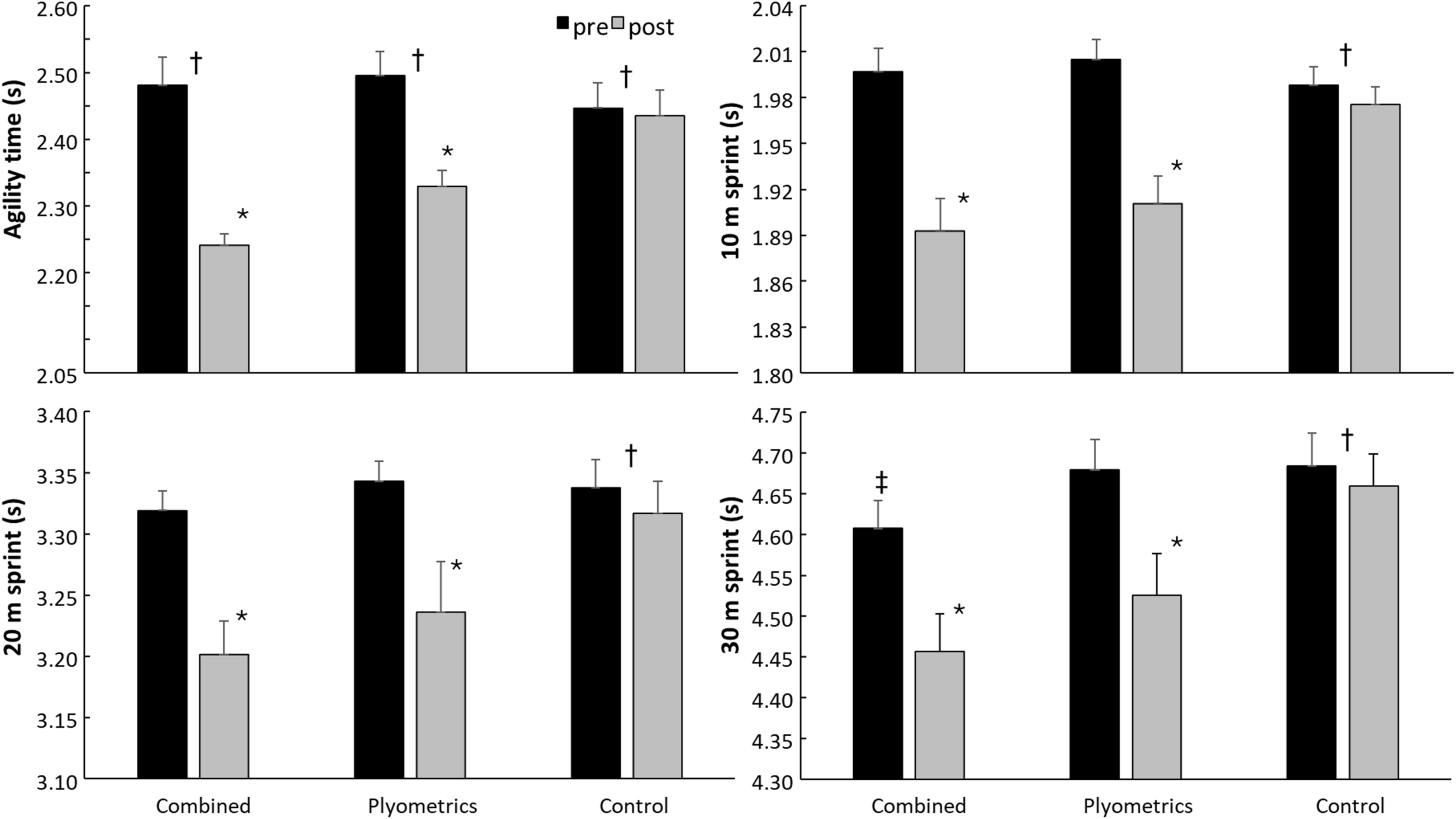
Mean (95% CI) agility and sprint times at 10, 20 and 30 m at pre and posttest for each group. * indicates a significant difference with the pretest. † Indicates a significant difference with the other two groups from pre – to post test. ‡ Indicates a significant difference with the other two groups at the pretest.

For baseline agility and sprint performance, no significant between-group differences were observed for most variables (p > 0.05), except for 30-m sprint time (F = 4.9, p = 0.012, η² = 0.19), for which the combined group was significantly faster than the other two groups. For agility and sprint performance, significant effects of time (F ≥ 43.7, p < 0.001, η² ≥ 0.50), time × group interaction (F ≥ 5.9, p ≤ 0.005, η² ≥ 0.30), and group (F ≥ 3.36, p < 0.044, η² ≥ 0.14) were found for all parameters. Holm-Bonferroni *post hoc* comparisons revealed that both the plyometric and combined training groups significantly improved their performance from pre- to post-test (3.2–5.2%), whereas the control group did not. Pairwise comparisons of pre-to-post changes showed small-to-large effect sizes. Larger effects were observed for both experimental groups compared with the control group (Cohen’s d = 1.58–3.27), whereas the effects for the combined versus plyometric comparisons were small for sprint performance (d = 0.03 to 0.23) and large for reactive agility (d = 0.83). In addition, reactive agility improved significantly more in the combined group than in the plyometric group (9.5% vs. 6.6%; **Figure 4**).

**Figure 5:**
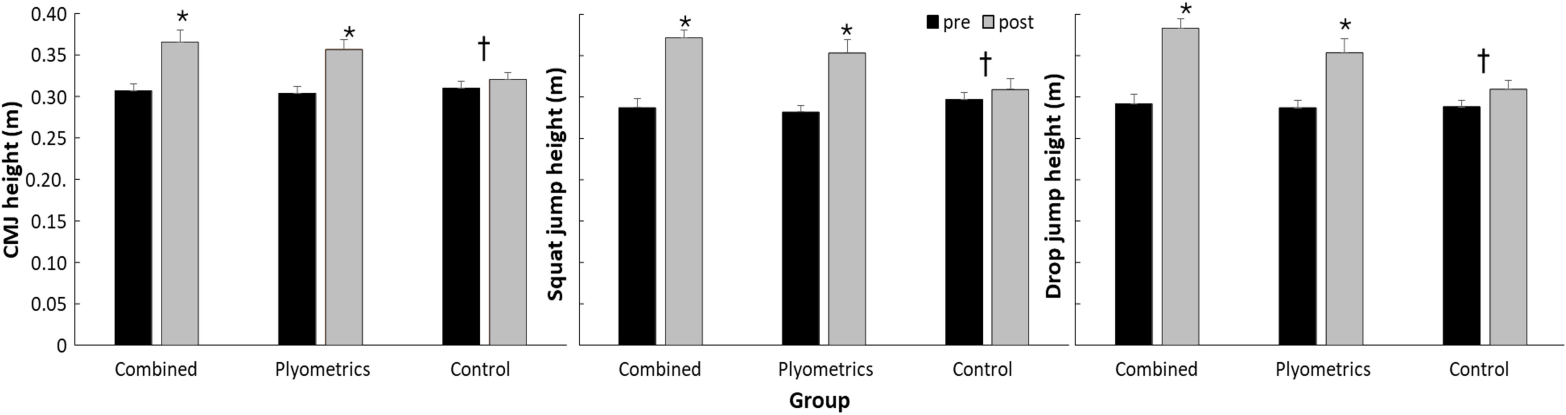
Mean (95% CI) jumping height at pre and posttest for each group of the counter movement jump (CMJ, squat jump and drop jump. * indicates a significant difference with the pretest. † Indicates a significant difference with the other two groups from pre- to post test.

For jump performance, *post hoc* comparisons revealed that both training groups increased squat jump, countermovement jump, and drop jump performance by 19–31%, with no significant differences between the two training groups, whereas the control group did not significantly change any jump performance over time. Pairwise comparisons of pre-to-post changes showed large effect sizes for both experimental groups compared with the control group (Cohen’s d = 1.38–2.89). In contrast, the effects for the combined versus plyometric comparisons were small-to-medium (d = 0.17–0.74), supporting the absence of significant between-group differences for jump performance (**Figure 5**).

**Figure 6:**
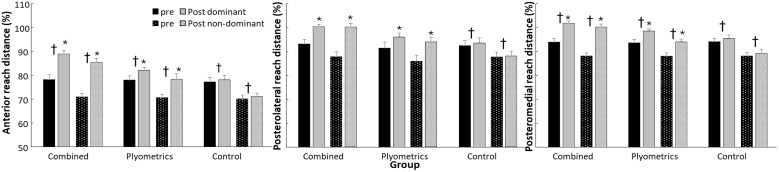
Mean (95% CI) reach distance related to leg length in anterior, posterolateral and posteromedial direction for dominant and non-dominant leg at pre and posttest for each group. * indicates a significant difference with the pretest. † Indicates a significant difference with the other two groups from pre- to post test.

For dynamic postural control, *post hoc* testing revealed that Y-Balance Test performance significantly increased from pre- to post-test in both training groups for both dominant and non-dominant legs in all three directions, whereas the control group did not significantly change in any direction. However, the combined group improved dynamic postural control significantly more than the plyometric group in the anterior and posteromedial directions for the dominant leg (17.6–14.8% vs. 6.6–7.2%) and non-dominant leg (14.2–11.6% vs. 8.2–6.2%). Pairwise comparisons of pre-to-post changes showed medium-to-large effect sizes for the combined versus plyometric comparisons (Cohen’s d = 0.54–2.16), with larger effects particularly observed in the anterior and posteromedial directions for both limbs (d = 1.12–2.16). Large effects were also observed for both experimental groups compared with the control group (d = 0.96–5.3; **Figure 6**).

**Figure 7:**
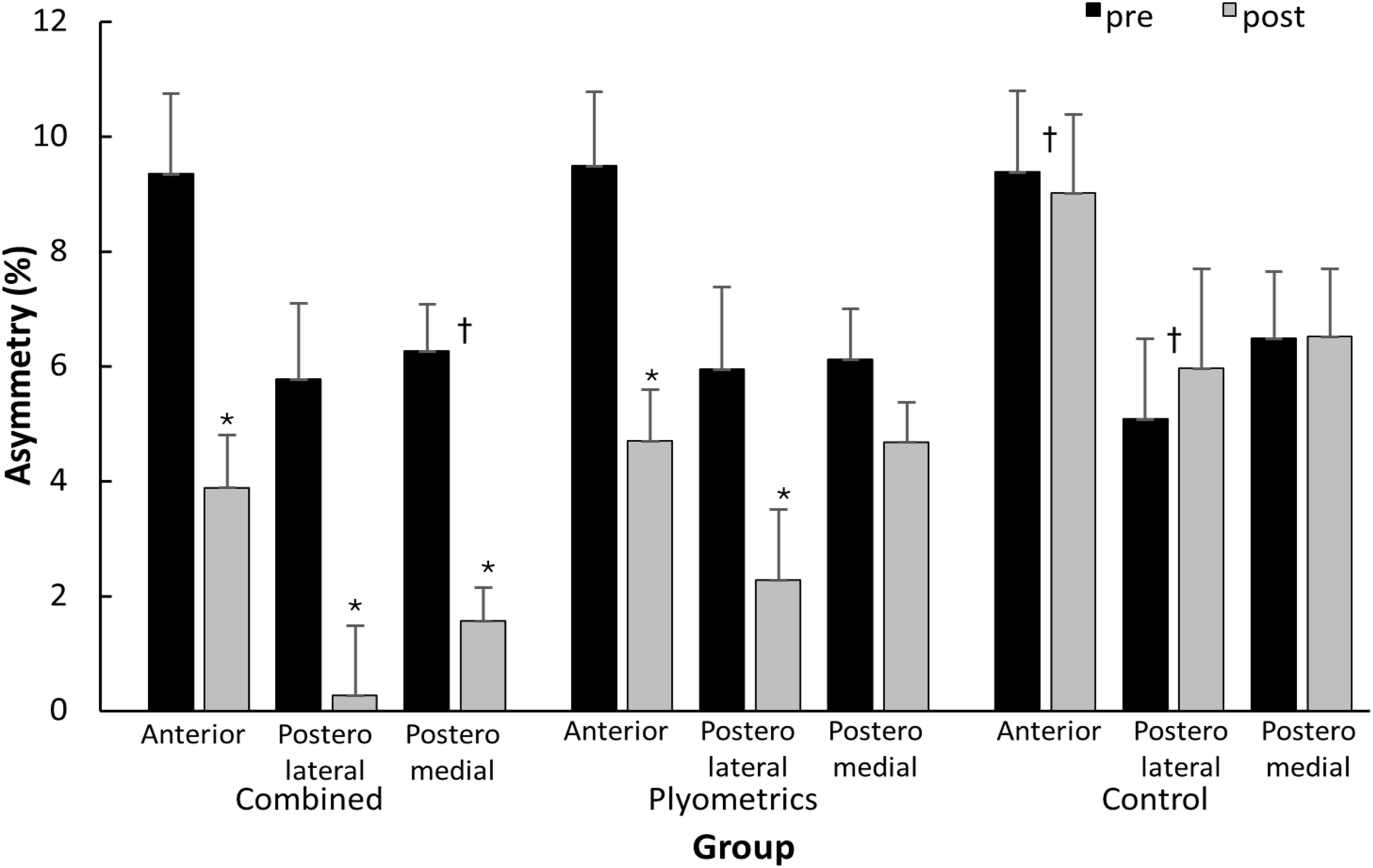
Mean (95% CI) asymmetry between legs in the three different directions at pre and posttest for each group. * indicates a significant difference with the pretest. † Indicates a significant difference with the other two groups from pre- to post test.

Inter-limb asymmetry decreased significantly in all three directions for the combined group, whereas it decreased significantly only in the anterior and posterolateral directions for the plyometric group. No significant changes in asymmetry were found in any direction for the control group. Consequently, a significantly greater decrease in posteromedial asymmetry was observed in the combined group compared with the other two groups (4.6% vs. 1.6% and −0.04%). Pairwise comparisons of pre-to-post changes showed small-to-large effect sizes, with larger effects observed for the combined group compared with the control group across the three directions (Cohen’s d = 1.58–2.36). The largest effect for the combined versus plyometric comparison was observed in the posteromedial direction (d = 1.50), supporting the greater reduction in asymmetry in this direction after combined training (**Figure 7**).

**Figure 8:**
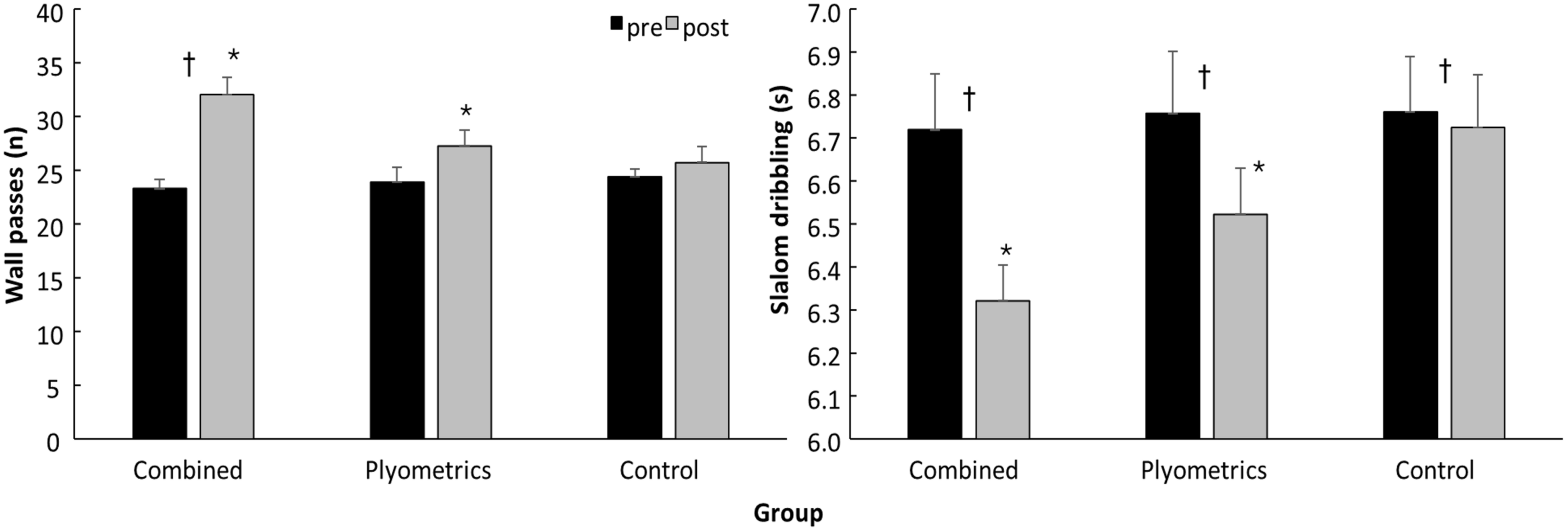
Mean (95% CI) wall passes and slalom dribble at pre and posttest for each group. * indicates a significant difference with the pretest. † Indicates a significant difference with the other two groups from pre – to post test.

*Post hoc* comparisons revealed significant improvements in sport-specific motor skills in both training groups, whereas the control group showed no significant changes. Moreover, the combined group demonstrated the greatest improvements from pre- to post-test. Pairwise comparisons of pre-to-post changes showed medium-to-large effect sizes, with larger effects observed for the combined group compared with the control group for both slalom dribble and wall-pass performance (Cohen’s d = 3.18–3.22). The combined group also showed larger effects than the plyometric group for both sport-specific tests (d = 0.87–1.84). However, in the wall-pass test, no significant difference in improvement was observed between the plyometric group and the control group with a medium effect size (d = 0.69; **Figure 8**).

## 4 Discussion

The present study examined the effects of combining multidirectional plyometric training with balance training performed on an unstable surface on neuromuscular performance, dynamic postural control, sport-specific motor skills, and inter-limb balance asymmetry in U17 soccer players. The main findings indicate that both experimental interventions were effective in enhancing several performance-related outcomes when compared with regular soccer training alone. However, the combined training program conferred additional benefits in selected variables, particularly reactive agility, dynamic postural control in specific reach directions, inter-limb balance asymmetry, and sport-specific skills. The present study extends previous work by showing that adding unstable-surface balance exercises to a multidirectional plyometric program may provide additional benefits beyond plyometric training alone in youth soccer players.

Regarding neuromuscular performance, both experimental groups showed significant improvements relative to the control group, thereby supporting previous evidence on the effectiveness of plyometric training for enhancing explosive strength and speed-related abilities in youth athletes (Chelly et al., 2010; Markovic and Mikulic, 2010; Ramírez-Campillo et al., 2014). Plyometric training is known to improve stretch-shortening cycle efficiency, motor-unit recruitment, neural drive, and intermuscular coordination, which may enhance force production during explosive movements such as jumping and sprinting (Miller et al., 2006; Markovic and Mikulic, 2010). In the present study, both training groups improved sprint and jump performance, whereas the control group did not exhibit meaningful changes. Moreover, no significant between-group differences were observed for the jump variables, suggesting that the shared plyometric component was the principal stimulus underlying the gains in lower-limb explosive performance. This interpretation is supported by previous studies reporting improvements in jumping and sprinting after plyometric training in soccer players and team-sport athletes (Chelly et al., 2010; Ramírez-Campillo et al., 2014; Kons et al., 2023).

Sprint performance also improved significantly in both training groups. These findings agree with previous studies showing that plyometric training can enhance sprinting ability by improving lower-limb power output and the efficiency of force application during acceleration (Chelly et al., 2010; Ramírez-Campillo et al., 2014). However, the additional balance training did not result in a clear superiority of the combined group across all sprint distances. This may be because linear sprinting depends strongly on horizontal force production, acceleration mechanics, and stretch-shortening cycle efficiency, which were targeted by the plyometric component in both experimental groups. By contrast, reactive agility improved significantly more in the combined group than in the plyometric group, suggesting that the addition of unstable-surface balance exercises may have enhanced the players’ ability to regulate posture and rapidly reorganize movement in response to an external stimulus.

This interpretation is supported by the nature of unstable-surface training. Such exercises increase proprioceptive demands and require continuous postural adjustments, thereby promoting sensorimotor integration and neuromuscular coordination (Gruber and Gollhofer, 2004; Behm and Colado, 2012). These adaptations may improve the activation of stabilizing musculature and the control of body position during rapid directional changes, which could partly explain the greater improvement in reactive agility observed in the combined training group. Comparable observations have been reported in previous studies investigating neuromuscular and balance training interventions in young athletes (Lesinski et al., 2015; Granacher et al., 2016).

A further important finding of the present study was the significant improvement in dynamic postural control following the intervention, with greater gains in the combined training group. Although both training groups improved their Y-Balance performance, the combined group showed significantly greater improvements than the plyometric group in the anterior and posteromedial directions for both the dominant and non-dominant legs. These findings are consistent with earlier reports showing that balance training on unstable surfaces can enhance proprioception, sensorimotor control, and postural stability (Paillard et al., 2006; Zech et al., 2010). Such adaptations are especially relevant in soccer, where players are repeatedly required to perform unilateral actions, including kicking, landing, and changing direction under dynamic conditions.

The asymmetry findings further reinforce the potential value of the combined intervention. Inter-limb asymmetries have been associated with impaired athletic performance and an elevated injury risk in athletes (Bishop et al., 2018b; Brown et al., 2018; Dos’ Santos et al., 2018). In the present study, asymmetry decreased significantly in all three directions in the combined group, whereas the plyometric group showed significant reductions only in the anterior and posterolateral directions. This greater reduction in the combined group may be explained by the specific unilateral postural demands of unstable-surface balance exercises. These exercises require continuous adjustments of the stance limb, enhanced proprioceptive feedback, and coordinated activation of stabilizing musculature around the ankle, knee, and hip. When combined with multidirectional plyometric exercises, these adaptations may have improved the ability of both limbs to control movement in different directions and may have promoted more symmetrical lower-limb function. Therefore, the observed reductions in asymmetry are likely related not only to gains in explosive performance but also to improved sensorimotor control and postural regulation.

Both experimental groups also improved sport-specific motor skills, whereas the control group did not. This suggests that the observed physical adaptations were accompanied by meaningful enhancements in technical performance. The greater gains seen in the combined group in the sport-specific tasks may be explained by the combined effects of enhanced neuromuscular performance and improved postural control, which together may facilitate more efficient movement execution and motor coordination during sport-specific actions (Gonzalo-Skok et al., 2017; Paillard, 2023). Nevertheless, these benefits were not identical across all skill-based tests. In particular, the wall-pass test did not differ significantly between the plyometric and control groups. This may be because passing performance depends not only on lower-limb power but also on technical proficiency, perceptual-motor control, ball familiarity, and task-specific practice. Thus, plyometric training alone may not have provided a sufficiently specific stimulus to induce greater improvements in this technical skill than regular soccer training.

The lack of significant pre-to-post changes in the control group for most variables also deserves consideration. Regular soccer training alone may maintain physical and technical capacities during the intervention period, but it may not provide a sufficiently targeted or progressive neuromuscular stimulus to induce measurable improvements in jumping, sprinting, dynamic balance, asymmetry, or reactive agility over eight weeks. This finding supports the practical value of structured complementary training programs, such as multidirectional plyometric training and combined plyometric-balance training, when the objective is to improve specific physical and functional qualities in youth soccer players.

From a practical standpoint, an interesting aspect of the present findings is that the combined group achieved superior adaptations in some balance-, agility-related and sport-specific skill variables despite performing a lower plyometric contact volume than the plyometric-only group, since part of the training session was devoted to unstable-surface balance exercises. This may indicate that the inclusion of balance training enhances the specificity and efficiency of the training stimulus rather than merely increasing training load. Accordingly, integrating unstable-surface balance exercises within plyometric sessions may be an effective strategy for simultaneously improving several important physical and functional qualities in youth soccer players.

Despite the promising findings, several limitations should be acknowledged. First, the sample included only male U17 soccer players, which limits the generalizability of the results to other age categories and to female players. Second, the intervention lasted only eight weeks, and therefore the long-term effects of combined plyometric and balance training remain unclear. Third, although sport-specific motor skills were assessed, match-related performance variables were not included. Fourth, the total training stimulus differed between groups because the combined group performed balance exercises in addition to the plyometric component, whereas the plyometric group performed only plyometric training and the control group continued regular soccer training. Therefore, it is not possible to fully separate the effects of training content from differences in overall training stimulus. Fifth, detailed information on previous exposure to structured plyometric or balance training was not collected, which may have influenced individual responsiveness to the intervention. Future studies should examine the long-term effects of combined multidirectional plyometric and unstable-surface balance training in different populations and determine whether these improvements translate into enhanced match performance and reduced injury risk.

## 5 Conclusion

In conclusion, the present findings indicate that both multidirectional plyometric training alone and combined multidirectional plyometric training with balance exercises performed on an unstable surface are effective for improving neuromuscular, postural, and sport-specific performance in U17 soccer players. However, the combined training approach appears to provide additional benefits for reactive agility, selected measures of dynamic postural control, inter-limb balance asymmetry and sport-specific skills. From a practical perspective, incorporating balance exercises performed on an unstable surface into a multidirectional plyometric training program may represent an effective strategy for optimizing functional adaptations in youth soccer players. Such an approach may be particularly relevant during preseason and player development phases, when the objective is to improve explosive performance, postural control, and movement symmetry within a structured conditioning program.

## Data Availability

The raw data supporting the conclusions of this article will be made available by the authors, without undue reservation.
